# State-Level and County-Level Estimates of Health Care Costs Associated with Food Insecurity

**DOI:** 10.5888/pcd16.180549

**Published:** 2019-07-11

**Authors:** Seth A. Berkowitz, Sanjay Basu, Craig Gundersen, Hilary K. Seligman

**Affiliations:** 1 Division of General Medicine and Clinical Epidemiology, Department of Medicine, University of North Carolina at Chapel Hill School of Medicine, Chapel Hill, North Carolina; 2 Cecil G. Sheps Center for Health Services Research, University of North Carolina at Chapel Hill, Chapel Hill, North Carolina; 3 Research and Analytics, Collective Health, San Francisco, California; 4 School of Public Health, Imperial College London, London, United Kingdom; 5 Center for Primary Care, Harvard Medical School, Boston, Massachusetts; 6 Department of Agricultural and Consumer Economics, University of Illinois, Urbana, Illinois; 7 Departments of Medicine and Epidemiology and Biostatistics, University of California San Francisco, San Francisco, California; 8 UCSF Center for Vulnerable Populations at Zuckerberg San Francisco General Hospital, San Francisco, California

## Abstract

**Introduction:**

Food insecurity, or uncertain access to food because of limited financial resources, is associated with higher health care expenditures. However, both food insecurity prevalence and health care spending vary widely in the United States. To inform public policy, we estimated state-level and county-level health care expenditures associated with food insecurity.

**Methods:**

We used linked 2011–2013 National Health Interview Survey/Medical Expenditure Panel Survey data (NHIS/MEPS) data to estimate average health care costs associated with food insecurity, Map the Meal Gap data to estimate state-level and county-level food insecurity prevalence (current though 2016), and Dartmouth Atlas of Health Care data to account for local variation in health care prices and intensity of use. We used targeted maximum likelihood estimation to estimate health care costs associated with food insecurity, separately for adults and children, adjusting for sociodemographic characteristics.

**Results:**

Among NHIS/MEPS participants, 10,054 adults and 3,871 children met inclusion criteria. Model estimates indicated that food insecure adults had annual health care expenditures that were $1,834 (95% confidence interval [CI], $1,073–$2,595, *P* < .001) higher than food secure adults. For children, estimates were $80 higher, but this finding was not significant (95% CI, −$171 to $329, *P* = .53). The median annual health care cost associated with food insecurity was $687,041,000 (25th percentile, $239,675,000; 75th percentile, $1,140,291,000). The median annual county-level health care cost associated with food insecurity was $4,433,000 (25th percentile, $1,774,000; 75th percentile, $11,267,000). Cost variability was related primarily to food insecurity prevalence.

**Conclusions:**

Health care expenditures associated with food insecurity vary substantially across states and counties. Food insecurity policies may be important mechanisms to contain health care expenditures.

**Summary**

**What is already known on this topic?**

Food insecurity is associated with higher health care costs, on average.

**What is added by this report?**

We found substantial variation in state- and county-level health care expenditures associated with food insecurity. We also found that higher food insecurity prevalence is more strongly associated with higher spending than differences in health care prices or intensity of health care use.

**What are the implications for public health practice?**

A multi-level strategy that encompasses both area-level determinants of food insecurity (eg, local labor market factors and state-earned income tax credits) and hunger safety net programs may improve public health.

## Introduction

Food insecurity, or uncertain access to food because of limited financial resources, affected 12.9% of Americans in 2016 — more than 40 million individuals (1). Food insecurity is associated with numerous chronic health conditions, including diabetes mellitus, hypertension, coronary heart disease, chronic kidney disease, and depression (2–6). Perhaps for this reason, estimates from both the United States and Canada indicate that, on average, health care costs are substantially higher among food-insecure individuals than among food-secure individuals (7–9).

Although food insecure individuals in the United States experience higher health care costs on average, this average likely obscures substantial variation across states and counties. The Map the Meal Gap study (http://map.feedingamerica.org/) has shown that US food insecurity rates vary widely (10). Similarly, local pricing and intensity of health care use also differ in the United States, resulting in widespread variation in health care spending (11,12). Furthermore, these patterns do not necessarily match; an area with higher food insecurity may have lower health care prices, and vice versa. This means that estimating local health care costs associated with food insecurity is not straightforward.

Understanding variation in health care costs associated with food insecurity has substantial public health implications, because doing so can inform the implementation of new initiatives (eg, the Centers for Medicare & Medicaid Services’ Accountable Health Communities Model [13]) or state and local public health and nutrition programs. Such programs could focus scarce resources on areas where health care costs associated with food insecurity are high. Furthermore, local economic policy, particularly state-earned income tax credits, local wage conditions, and housing policies can influence food insecurity (14,15). Therefore, understanding variations in health care costs associated with food insecurity has implications beyond public health.

To help inform both policies and programs to address these issues, we sought to estimate county-level and state-level health care costs associated with food insecurity in the United States.

## Methods

### Study design and data sources

To generate local estimates of health care costs associated with food insecurity, we needed 3 key pieces of information: 1) the mean per-person dollar amount of excess health care expenditures among adults and children; 2) the number of food-insecure adults and children residing in each county and state; and 3) the variation, from the national average, in health care costs for each county and state. The rationale for this was that, because health care expenditures exhibit substantial geographic variability, a similar individual might have lower health care costs if they resided in a low-cost area (in terms of health care prices) and higher health care costs if they lived in a high-cost area, even if their health care needs were exactly the same. Because no single data source had information on all 3 of these factors, we needed to combine data from several sources to generate our estimates. The institutional review board at the University of North Carolina at Chapel Hill exempted this analysis of secondary data from human subjects review.

### National Health Interview Survey/Medical Expenditure Panel Survey

To estimate the excess health care costs, if any, associated with food insecurity, we used linked data from the National Health Interview Survey (NHIS) (16) and the Medical Expenditure Panel Survey (MEPS) (17). NHIS is a nationally representative epidemiologic surveillance survey of the civilian noninstitutionalized US population (16). MEPS is a nationally representative cohort that collects detailed data on health care expenditures over a 2-year period and is drawn from NHIS participants (17). We used data collected from NHIS participants in 2011 who participated in MEPS during 2012–2013. We extracted information on the exposure of food security status from NHIS, which used a 10-item version of the United States Department of Agriculture food security survey module for adults with a 30-day look-back window (16). In accordance with standard scoring, raw scores of 0 to 2 were considered food secure and raw scores of 3 to 10 were considered food insecure (16). We used the MEPS total health care expenditures variable, which includes all health care costs (eg, inpatient admissions, outpatient visits, medication costs). Using NHIS food insecurity data and MEPS health care cost data ensures appropriate time ordering between the hypothesized exposure and outcome. More details on NHIS and MEPS data, as well as on the estimates and statistical methods used in this study, are provided at https://saberkowitz.web.unc.edu/supplemental-information/state-and-local-healthcare-costs/.

### Map the Meal Gap

Data on the prevalence of food insecurity among adults and children at the county and state level came from Map the Meal Gap (MMG), which is based on US Census data (including the American Community Survey and Current Population Survey) and Bureau of Labor Statistics data. MMG methods have been published (10). MMG uses a 2-step process established by Feeding America to obtain estimates of food insecurity prevalence for all US counties. In the first step, the state-level determinants of food insecurity (for both children and adults) are estimated based on data from 2001 through 2016. The model components used are unemployment, poverty, median income, percentage Hispanic ethnicity, percentage African-American race, percentage living in owned housing, year fixed effects, and state fixed effects. These models are then used in the second step to produce food insecurity estimates at the county level, using county-specific variables. For our study, the county-specific variables were drawn from the 2016 American Community Survey 5-year estimates.

### Dartmouth Atlas of Health Care

To estimate how a given county or state differed in health care spending (either based on prices or intensity of care) from the national average, we used data from the Dartmouth Atlas of Health Care (www.dartmouthatlas.org/), covering 2012–2013 because that was when cost data were collected, to calculate a “cost factor.” The resulting cost factor is greater than 1 for areas with higher-than-average costs and less than 1 for areas with lower-than-average costs (18). 

### Statistical analysis

Step 1 of our analysis was to determine national estimates of excess health care costs, if any, associated with food insecurity. To do this, we used NHIS and MEPS data. Analyses incorporated representativeness weights and survey design (clustering) information as appropriate. Because the mechanisms through which food insecurity may be associated with health care costs are likely different between adults and children, we stratified our data by age (≥18 years for adults and <18 years for children) and then made separate estimates in these groups. To generate the cost estimates, we drew on prior work examining the association between food insecurity and health care costs (8). Because health care cost data are notoriously difficult to analyze (19) and generalized linear models rely on certain assumptions that may not always be met in practice, we applied a targeted maximum likelihood estimation approach (TMLE). TMLE is a doubly robust analytic strategy that initially creates an estimate of the excess health care costs associated with food insecurity and then updates that estimate using a submodel that estimates the probability of being food insecure (20).

Using NHIS and MEPS data allowed us to estimate the mean per-person cost associated with food insecurity for adults and children, but NHIS and MEPS are not designed to estimate health care costs for every county or state. Therefore, in step 2, we multiplied our nationally representative per-person estimate of health care costs by the number of food insecure adults and children in each county and state (using data from MMG). Then, to account for county and state differences in health care spending, we multiplied by the cost factor for the locality. To bound the uncertainty in the estimates, we created a lower and upper bound by using the 95% confidence interval (CI) for the NHIS/MEPS estimate of average health care costs associated with food insecurity. Finally, we conducted correlation analyses to help understand whether local variations in health care costs associated with food insecurity are more closely related to food insecurity prevalence or local health care spending characteristics.

All dollar estimates were inflation adjusted to December 2016 dollars, following MEPS guidance (https://meps.ahrq.gov/about_meps/Price_Index.shtml). All analyses — with the exception of MMG estimates, which were derived using Stata version 14.2 (StataCorp LP) — were conducted in SAS version 9.4 (SAS Institute, Inc) and R version 3.4.2 (R Foundation).

## Results

In the analyses of health care costs associated with food insecurity, 10,054 adults and 3,871 children were included. Both food-insecure adults and children were more likely than their food-secure counterparts to be racial/ethnic minorities, have lower income, and lack health insurance (Table 1).

**Table 1:** Demographic Characteristics of Participants, Medical Expenditure Panel Survey, United States, 2012–2013^a^

Characteristic	Adults			Children		
	Food Secure (n = 8,306)	Food Insecure (n = 1,748)	*P* Value^b^	Food Secure (n = 3,017)	Food Insecure (n = 854)	*P* Value^b^
**Mean age (SE), y**	44.9 (16.9)	41.2 (15.3)	<.001	8.8 (4.8)	9.0 (4.8)	.67
**Female**	50.9 (4,398)	53.9 (1,003)	.02	49.6 (1,483)	49.8 (418)	.92
**Race/ethnicity**						
Non-Hispanic white	69.6 (3,561)	56.3 (480)	<.001	58.3 (869)	42.0 (149)	<.001
Non-Hispanic black	10.3 (1,561)	18.3 (498)		13.4 (625)	21.5 (252)	
Hispanic	12.7 (2,209)	22.4 (697)		21.3 (1,236)	32.9 (423)	
Asian/multi/other	7.4 (975)	3.1 (73)		7.0 (287)	3.6 (30)	
**Health insurance**						
Private	69.6 (4,956)	36.4 (463)	<.001	66.1 (1,452)	32.0 (166)	<.001
Medicare	8.9 (690)	10.3 (187)		NA	NA	
Other public	6.6 (893)	18.2 (427)		27.7 (1,294)	55.3 (565)	
Uninsured	14.9 (1,767)	35.1 (671)		6.3 (258)	12.7 (123)	
**Education**						
<High school diploma	11.7 (1,495)	24.3 (595)	<.001	NA	NA	NA
High school diploma	24.4 (2,159)	33.1 (540)		NA	NA	
>High school diploma	63.9 (4,652)	42.6 (613)		NA	NA	
**Income, % of federal poverty level**						
<100	9.8 (1,242)	34.1 (764)	<.001	15.6 (866)	43.2 (474)	<.001
100–199	14.9 (1,593)	34.1 (561)		19.3 (727)	33.9 (256)	
≥200	75.3 (5,471)	31.9 (423)		65.1 (1,424)	22.9 (124)	
**Resides in nonmetropolitan area**	13.7 (964)	16.8 (232)	.16	14.4 (372)	18.2 (124)	.21
**Census region**						
Northeast	17.7 (1,423)	16.7 (274)	.41	15.7 (454)	13.1 (127)	.58
Midwest	22.6 (1,514)	21.2 (304)		22.3 (591)	22.5 (149)	
South	36.3 (3,013)	41.1 (718)		38.1 (1,053)	42.9 (346)	
West	23.5 (2,356)	21.0 (452)		23.9 (919)	21.5 (232)	

Abbreviations: NA, not applicable; SE, standard error.

a Values are % (N), unless otherwise indicated. Percentages were weighted to be nationally representative.

b *P* values were determined by *t* test for continuous variables and χ^2^ test for categorical variables and incorporated Medical Panel Expenditure Survey weights and clustering information. Significance testing was conducted by using survey weight and design information.

In TMLE analyses that accounted for age, sex, race/ethnicity, income, education, health insurance, metropolitan residence, and region of residence within the country, model-based estimates showed that adults who were food insecure had annual health care expenditures that were $1,834 (95% CI, $1,073–$2,595) higher than adults who were food secure (*P* < .001). In children, the model-based estimate for health care costs associated with food insecurity was $80 annually, but this finding was not significant (*P* = 0.53, 95% CI, −$171 to $329). Among approximately 28,266,000 food-insecure adults and 12,938,000 food-insecure children in the United States in 2016, using these model-based point estimates of the excess cost associated with food insecurity translates to approximately $52.9 billion in excess health care expenditures associated with food insecurity in 2016 (95% CI, $31.8 billion to $74.3 billion). This represents 3% to 6% of the approximately $1.2 trillion in annual health care expenditures we estimate from MEPS data. Because the estimate for children was not significantly different from $0, taking only adult costs yielded a national estimate of $51.8 billion in excess health care expenditures in 2016 (95% CI, $31.7 billion to $74.2 billion).

**Figure 1:**
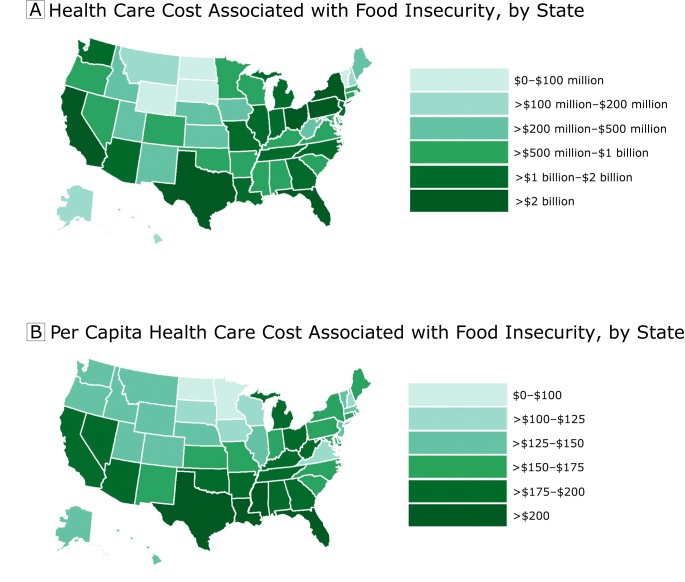
Health care costs associated with food insecurity (A) and per capita health care costs associated with food insecurity (B), by state, United States, 2012–2013.

**Figure 2:**
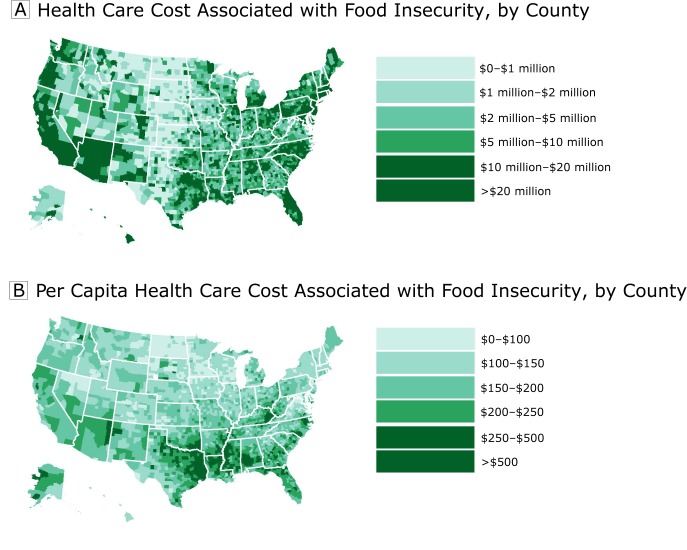
Health care costs associated with food insecurity (A) and per capita health care costs associated with food insecurity (B), by county, United States, 2012–2013.

Using the model-based estimates from our main analyses (eg, point estimate for adults of $1,834), we then calculated the costs associated with food insecurity for each state (including the District of Columbia) and county in the United States (Figures 1 and 2). Estimates by state are presented in Table 2 and estimates by county are presented in the Appendix. At the state level, adult food insecurity prevalence ranged from 6.8% (North Dakota) to 17.6% (Mississippi), and child food insecurity prevalence ranged from 10.3% (North Dakota) to 25.0% (New Mexico). At the state level, the mean annual model-based health care cost associated with food insecurity was $1,087,815,000 (standard deviation [SD], $1,407,496,000), and the median annual health care cost associated with food insecurity was $687,041,000 (25th percentile, $239,675,000; 75th percentile, $1,140,291,000). The state with the highest annual model-based health care cost associated with food insecurity was California, at $7,213,940,000, and the state with the lowest annual health care cost associated with food insecurity was North Dakota at $57,587,000. On a per capita basis, Mississippi had the highest health care cost associated with food insecurity, while North Dakota had the lowest. The 5 states with the highest per capita health care costs associated with food insecurity were Mississippi, Texas, Louisiana, Florida, and Oklahoma. The mean annual county-level health care cost associated with food insecurity was $17,905,000 (SD, $69,194,000), and the median annual county-level health care cost associated with food insecurity was $4,433,000 (25th percentile, $1,774,000; 75th percentile, $11,267,000).

**Table 2:** Estimates of Health Care Costs Associated With Food Insecurity, by US State, Using Food Insecurity Prevalence, United States, 2016 from Map the Meal Gap Data

State	No. of Food Insecure Adults (%)	No. of Food Insecure Children (%)	State Cost Factor	Estimated State Cost, Thousand $	Lower Bound of Estimated State Cost, Thousand $	Upper Bound of Estimated State Cost, Thousand $	Estimated State Cost Per Capita^a^, $
AK	64,480(11.7)	33,884 (18.1)	0.8789	106,318	55,716	156,860	144
AL	560,070(15.0)	253,580 (22.9)	0.9128	956,117	508,971	1,402,800	197
AR	343,840(15.2)	164,080 (23.2)	0.8763	564,100	298,715	829,197	190
AZ	733,880(14.4)	362,720 (22.4)	0.9581	1,317,343	695,033	1,938,958	196
CA	3,472,760(11.8)	1,727,632 (18.9)	1.1086	7,213,940	3,803,437	10,620,612	187
CO	417,230(10.1)	185,223 (14.9)	0.8738	681,579	363,514	999,321	127
CT	283,170(10.1)	116,675 (15.1)	1.0798	570,856	306,544	834,915	159
DC	57,280(10.5)	24,725 (21.6)	0.9490	101,571	54,315	148,780	154
DE	73,940(10.1)	32,350 (15.8)	0.9989	138,042	73,725	202,295	148
FL	2,039,750(12.9)	835,693 (20.6)	1.1155	4,247,553	2,282,032	6,211,211	213
GA	1,035,370(13.6)	529,473 (21.2)	0.9193	1,784,569	938,066	2,630,099	177
HI	129,110(11.7)	54,653 (17.7)	0.7604	183,378	98,236	268,438	130
IA	247,770(10.4)	116,656 (16.0)	0.8118	376,466	199,629	553,113	121
ID	145,630(12.1)	71,196 (16.5)	0.8116	221,389	116,940	325,723	135
IL	995,450(10.1)	476,810 (15.9)	1.0275	1,915,055	1,013,714	2,815,415	149
IN	605,750(12.1)	282,367 (17.8)	0.9632	1,091,820	579,544	1,603,553	166
KS	267,000(12.3)	137,739 (19.1)	0.8933	447,273	234,882	659,418	154
KY	467,210(13.8)	191,223 (18.9)	0.9800	854,718	459,245	1,249,815	194
LA	512,920(14.5)	249,267 (22.4)	1.0621	1,020,292	539,269	1,500,786	220
MA	416,760(7.8)	161,325 (11.6)	1.1329	880,540	475,361	1,285,353	131
MD	455,340(9.9)	198,685 (14.7)	1.0878	925,705	494,519	1,356,459	155
ME	131,700(12.3)	51,332 (19.8)	0.8540	209,780	113,186	306,287	158
MI	919,310(12)	354,363 (15.9)	1.0507	1,801,282	972,763	2,629,056	182
MN	351,350(8.4)	169,770 (13.2)	0.8265	543,802	287,595	799,728	100
MO	611,280(13.1)	247,281 (17.7)	0.9180	1,047,318	563,303	1,530,879	173
MS	397,610(17.6)	172,578 (23.6)	0.9756	724,893	387,435	1,062,014	243
MT	93,310(11.7)	40,025 (17.8)	0.7634	133,085	71,208	194,901	130
NC	1,053,660(13.8)	473,482 (20.7)	0.8799	1,733,659	923,553	2,542,932	174
ND	38,440(6.8)	17,223 (10.3)	0.8012	57,587	30,687	84,460	78
NE	155,490(11)	83,025 (17.8)	0.8840	257,961	134,937	380,837	137
NH	82,530(7.8)	31,552 (11.8)	0.9171	141,127	76,266	205,931	106
NJ	631,780(9.1)	265,881 (13.2)	1.1356	1,339,957	718,193	1,961,117	150
NM	243,150(15.4)	125,202 (25.0)	0.7915	360,887	189,557	532,019	173
NV	263,100(12.1)	135,915 (20.4)	1.0504	518,266	272,122	764,124	183
NY	1,615,580(10.4)	740,983 (17.5)	1.1254	3,401,243	1,808,303	4,992,516	173
OH	1,188,300(13.3)	533,658 (20.2)	1.0077	2,239,144	1,192,906	3,284,307	193
OK	428,450(14.7)	210,461 (22.1)	0.9554	766,818	404,839	1,128,394	198
OR	397,650(12.7)	177,048 (20.6)	0.7844	583,165	310,938	855,115	146
PA	1,130,340(11.2)	456,719 (16.9)	1.0157	2,142,702	1,152,572	3,131,904	168
RI	89,170(10.6)	37,375 (17.6)	1.0114	168,426	90,306	246,471	160
SC	471,060(12.6)	200,828 (18.5)	0.8995	791,551	423,760	1,158,982	164
SD	65,720(10.2)	34,945 (16.7)	0.8096	99,845	52,253	147,380	117
TN	681,790(13.5)	296,567 (19.8)	0.9330	1,188,762	635,230	1,741,740	182
TX	2,937,940(14.8)	1,626,375 (22.8)	1.0894	6,011,628	3,131,262	8,888,450	223
UT	253,560(12.4)	142,565 (15.7)	0.8657	412,449	214,426	610,225	140
VA	610,550(9.5)	241,421 (12.9)	0.8687	989,503	533,241	1,445,347	119
VT	51,690(10.2)	18,811 (15.5)	0.8500	81,859	44,410	119,276	131
WA	639,870(11.7)	297,248 (18.5)	0.8654	1,036,145	550,179	1,521,596	146
WI	430,840(9.7)	215,845 (16.6)	0.8509	687,041	361,957	1,011,757	119
WV	186,350(12.7)	73,114 (19.2)	0.9408	327,036	176,354	477,581	177
WY	52,500(11.8)	24,108 (17.4)	0.8451	83,000	44,123	121,837	142

a Per capita refers to entire state population, not only to food insecure population within the state. Adult food insecurity prevalence represents number of food insecure adults in the state divided by the total number of adults, expressed as a percentage. Child food insecurity prevalence represents the number of food insecure children in the state, divided by the total number of children, expressed as a percentage. Prevalence estimates are from aggregated county estimates and may not exactly match official US Department of Agriculture state-level estimates derived from the Current Population Survey. The point estimate of health care costs associated with food insecurity in adults is $1,834 (95% confidence interval, $1,073–$2,595). Data source: Map the Meal Gap (10).

The components of our cost estimates were the number of food-insecure individuals and the cost factor that accounted for local care intensity and prices. We found that at both the county and state level, the number of individuals who were food insecure was strongly correlated with the total expenditure estimate (*r* ^2^ = 0.99 for county cost and *r* ^2^ = 0.99 for state cost). The total expenditure estimate was only weakly or moderately associated with the cost factor (*r* ^2^ = 0.22 for county cost and *r* ^2^ = 0.57 for state cost). This suggests that a high proportion of the variation in food insecurity–associated health care expenditures is attributable to the number of food-insecure individuals.

## Discussion

We found that food insecurity was associated with higher health care spending in adults and that this spending varied substantially across locality. Although patterns of local health care use and price explained some of this difference, the number of food-insecure individuals, and in particular the number of food-insecure adults, accounted for the largest share of variation in associated costs.

These findings are consistent with and expand our knowledge about the relationship between food insecurity and health care costs. Studies in both Canada (9) and the United States (7,8) have found that food insecurity is associated with higher health care costs. Specifically, a study from our research group (8) using similar methods found higher health care costs associated with food insecurity during a period when food insecurity prevalence was higher. Furthermore, recent research in the United States has found that, for several common clinical conditions, food insecurity is associated with excess health care costs even when accounting for other demographic and clinical characteristics (21). This may be related to several factors (2,22), including worse dietary quality in food-insecure individuals (23); trade-offs between food and other basics, such as medications, that make chronic disease management more difficult (24); and psychological factors, including stress and depressive symptoms (6). This study adds to this literature by quantifying the wide variation in excess expenditures. Although this study cannot determine why this variation occurs, the correlation analyses suggest that variation in model-based estimates of local health care costs associated with food insecurity is closely correlated with food insecurity prevalence in the area and less closely correlated with the local cost factor.

In our analyses, the point estimate for health care costs associated with food insecurity in children was small and not significantly different than $0. Although this study cannot determine why that is the case, past work suggests that food insecurity may be most closely related to increased health care cost through increased prevalence of chronic disease and exacerbation of those chronic conditions when they occur (8,22). If this is the case, then children may not see short-term (eg, the 2-year period in the NHIS/MEPS data) increases in health care costs simply because they are at low risk of developing these conditions, regardless of food security status. This does not imply, however, that food insecurity does not have long-term effects on children’s health or even short-term effects on important aspects of life that do not generate short-tern health care costs, like school achievement.

This study has implications for public health. Literature has demonstrated that local and state economic policies and conditions can have a substantial effect on food insecurity prevalence (14,15). In particular, lower tax burden (but not overall tax burden) for low-income individuals is associated with lower food insecurity, with strong associations between lower food insecurity and higher state-earned income tax credits (14,15). Other factors associated with lower food insecurity include local labor conditions and ease of access to hunger safety-net programs (14,15). For this reason, an important direction for future research will be to evaluate whether polices that reduce area food insecurity prevalence also lead to lower health care spending. Because there is evidence that individual-level nutrition interventions, particularly the Supplemental Nutrition Assistance Program (SNAP) (25–27) and medically tailored meal delivery programs (28), may also be associated with lower health care costs, having area-level policy options could provide a multilevel framework for addressing high health care spending by supporting access to proper nutrition. Fewer than 40% of individuals with food insecurity in this study had private health insurance, meaning that public health care programs, particularly at the state level, are shouldering much of the cost associated with food insecurity. SNAP and other nutrition programs are funded at the federal level, so if states worked to maximize uptake of federal nutrition programs, they may not only lower food insecurity rates but also decrease health care expenditures.

This study has several limitations. The costs estimated are likely conservative, because there is evidence that MEPS underestimates health care expenditures (29), and we did not consider indirect costs (like lost productivity owing to illness). Also, the sample used for estimating health care expenditures included only civilian noninstitutionalized individuals, which excludes some groups. The association between food insecurity and health care costs may not be fully related to food insecurity causing higher costs. There is likely to be a bidirectional relationship whereby food insecurity may worsen health, thus increasing health care costs, and worse health (and attendant expenses) may lead to food insecurity by decreasing the ability to work and increasing household debt. When examining the relationship between food insecurity and health care costs, there is a small delay between food security assessment in NHIS and the beginning of cost data collection in MEPS. Although assessment of food insecurity before collection of cost data is necessary to preserve time-ordering and mitigate reverse causation, the delay could lead to some misclassification (if food security status changes in the interval), which would tend to bias results to the null. Also, because NHIS and MEPS are not designed to yield county-level estimates of food insecurity prevalence or health care costs, we had to combine NHIS and MEPS data with data sources that were designed to provide more granular estimates. In addition, this is an inherently ecological analysis. However, since both the exposure (food insecurity prevalence) and the outcome (health care spending in the locality) were area-level assessments, this type of analysis is not subject to concerns about ecological fallacy (30). Finally, we did not have the ability to look at the specific distribution of comorbidities within each locality. To the extent that differences in comorbidities reflect differences in effect modifiers, actual local spending will not match the estimates. This would occur both for areas where individuals are less healthy than expected (and thus incur greater costs) and areas where individuals are healthier than expected (and correspondingly have lower health care costs).

Our study also has strengths. We used a nationally representative, longitudinal data set to estimate the association between food insecurity and health care costs. Furthermore, we used robust and well-validated methods to provide local estimates of food insecurity prevalence.

Food insecurity is associated with substantial health care expenditures, but there is evidence that this varies widely across states and counties. This variation suggests that local and state policies could be important mechanisms for improving health and containing health care expenditures. As health care cost containment remains a national priority, state and local strategies to reduce food insecurity rates may be an important public health tool.

## Appendix

Health Care Costs Associated With Food Insecurity in the United States, by County, 2012–2013. This appendix is available for download.
